# Thirty-day Outcomes of On-Pump and Off-Pump Coronary Artery Bypass Grafting: an Analysis of a Brazilian Sample by Propensity Score Matching

**DOI:** 10.21470/1678-9741-2021-0229

**Published:** 2022

**Authors:** Álvaro Rösler, Gabriel Constantin, Pedro Nectoux, Bruno Sell Holz, Estevan Letti, Marcela Sales, Fernanda Lucchese-Lobato, Fernando Lucchese

**Affiliations:** 1 Research Center of Cardiovascular Surgery, São Francisco Hospital, Santa Casa de Misericórdia de Porto Alegre, Porto Alegre, Rio Grande do Sul, Brazil.; 2 Pediatric brain-heart Clinic, Santo Antônio Hospital, Santa Casa de Misericórdia de Porto Alegre, Porto Alegre, Rio Grande do Sul, Brazil.

**Keywords:** Cardiopulmonary Bypass, Morbidity, Propensity Score, Logistic Model, Reoperation, Myocardial Infarction

## Abstract

**Introduction:**

Coronary artery bypass grafting (CABG) performed *with* and *without* cardiopulmonary bypass (CPB) support has been widely discussed in the literature. However, little is known about the outcomes of those techniques in Brazil. This study aims at exploring 30-day mortality and morbidity outcomes of on- and off-pump isolated CABG in a large sample from Southern Brazil.

**Methods:**

A single-center cohort with 1,767 patients undergoing isolated CABG (January 2013 - December 2018) was initially evaluated. Patients undergoing off-pump (N=397) and on-pump (N=1,370) CABG were identified. To obtain two completely homogeneous study groups, propensity score matching was used. The paired groups were compared by descriptive and univariate analyses. Then, logistic regression was used to verify the effects of on- and off-pump CABG on 30-day mortality.

**Results:**

None of the baseline characteristics showed significant difference between the groups (*P*>0.05). None of the analyzed morbidity outcomes showed any difference between the groups, including acute myocardial infarction (3.0% *vs*. 1.5%; *P*=0.192), stroke (2.4% *vs*. 4.2%; *P*=0.193), and major reoperation (0.6% *vs*. 0.3%; *P*=1.000), as well as the major adverse cardiovascular and cerebrovascular events composite outcome (6.3% *vs*. 7.5%; *P*=0.541). Mortality also did not differ (1.5% *vs*. 2.4%; *P*=0.401), and CPB support was not an independent predictor of risk for 30-day mortality (odds ratio: 2.052; 95% confidence interval: 0,609-6.913; *P*=0.246).

**Conclusion:**

After matching by propensity analyses, similar rates of on- and off-pump 30-day mortality and other major outcomes were observed. In addition, the use of CPB support was not an independent predictor of risk for the occurrence of 30-day mortality.

**Table:** 

Abbreviations, acronyms & symbols			
AF	= Atrial fibrillation	LV	= Left ventricle
AMI	= Acute myocardial infarction	MACCE	= Major adverse cardiovascular and cerebrovascular events
B	= Unstandardized regression weight.	NYHA	= New York Heart Association
CABG	= Coronary artery bypass grafting	OR	= Odds ratio
CI	= Confidence interval	PASP	= Pulmonary artery systolic pressure
COPD	= Chronic obstructive pulmonary disease	PCI	= Percutaneous intervention
CPB	= Cardiopulmonary bypass	PVD	= Peripheral vascular disease
CV	= Cardiovascular	RCTs	= Randomized clinical trials
EuroSCORE	= European System for Cardiac Operative Risk Evaluation	SE	= Standard error
HF	= Heart failure	STS	= Society of Thoracic Surgeons

## INTRODUCTION

In Brazil, coronary disease is a condition with very high prevalence^[1]^. The standard treatment for complex coronary disease is coronary artery bypass grafting (CABG)^[2-5]^. Coronary surgery was introduced in Brazil by Drs. Jatene and Zerbini in late 1960s and is the most widely performed surgical cardiovascular procedure in Brazil. In fact, more than 20,000 surgical revascularizations are performed every year, representing 113 CABGs per million inhabitants in Brazil annually^[1]^. CABG patients in Brazil have high prevalence of several cardiovascular risk factors, and the national mortality rates are about 6%^[6]^.

Worldwide, the question regarding the effectiveness of cardiopulmonary bypass (CPB) support has become debatable and even controversial. Randomized clinical trials (RCTs) comparing on and off CPB techniques have shown mixed results^[7-10]^. Thus, a scientific consensus is yet to be reached on the best practice for CPB support use in CABG^[12]^. Very few studies have compared both techniques in Brazilian samples, and no large-scale RCT has been done in Brazil with the same aim^[12-14]^.

In Brazil, controlled RCTs of cardiovascular surgeries are often not performed due to a dearth of clinical research teams in most cardiovascular centers, as well as lack of research funding. Recently, an emergent statistical methodology has been increasingly used to compare interventions by using observational data from cohorts. For instance, the propensity score matching analysis has become a feasible and powerful approach to study surgical outcome data^[10,15-17]^ without the costs of doing an RCT, while still controlling for heterogeneity in the sample.

This study aims at using a propensity score matching analysis to compare the outcomes of two groups of post isolated CABG patients (on- *vs*. off-pump) in a reference cardiovascular center in Southern Brazil.

## METHODS

This study protocol received full approval from the institutional ethics review board (2.006.177) and departmental research committee. It complies with the ethical guidelines of the Declaration of Helsinki. As this is a retrospective observational study of clinical surgical practice, the consent form was not required by the local committee.

We analyzed a single-center cohort with 1,767 patients who underwent isolated CABG between January 2013 and December 2018. Of these surgeries, 397 (22.5%) were performed with off-pump technique, and 1,370 (77.5%) were performed with on-pump technique. A standard median sternotomy was performed in all patients. As an uncontrolled cohort study, the criteria used to choose the surgical technique was subjective and dependent on each surgeon’s discernment.

Sample heterogeneity is often observed in randomized controlled trials, and this study used a propensity score matching analysis by a logistic regression model^[18]^ to obtain two completely homogeneous comparison groups. A logistic regression model was built with the categorical variable of CPB support as the dependent variable. The independent variables were 21 baseline and clinical characteristics including gender, age, weight, hypertension, diabetes, acute myocardial infarction (AMI), renal impairment, hemodialysis, smoking, chronic obstructive pulmonary disease (COPD), pulmonary artery systolic pressure (PASP), stroke, peripheral artery disease, atrial fibrillation (AF), New York Heart Association class III or IV heart failure, frailty, anemia, instable angina, previous cardiovascular surgery, stenosis > 50% in the left main coronary artery, and emergency surgery.

**Fig. 1:**
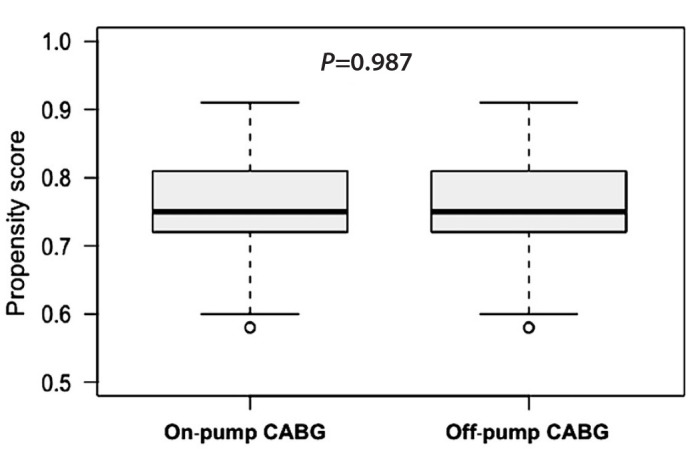
Boxplot of the propensity scores of stratified paired study groups. CABG=coronary artery bypass grafting.

The probabilities generated for each patient were used as scores to establish the best match. To form a pair, it was necessary to have the same value in the three first decimals. The fourth decimal being the tiebreaker criterion in the pairing. This way, it was possible to obtain 332 pairs (N=664) of very similar patients. In Figure 1, the equality of the propensity score matching values between the two intervention groups is presented.

After the propensity score matching, we performed normality analyses for all quantitative variables evaluated in the study - age, creatinine clearance, left ventricular ejection fraction, PASP, European System for Cardiac Operative Risk Evaluation (EuroSCORE) I, EuroSCORE II, and Society of Thoracic Surgeons (STS) score. The distribution pattern was evaluated by skewness and kurtosis coefficients and Kolmogorov-Smirnov test. Coefficients between-3 and +3 and a Kolmogorov-Smirnov test *P*-value > 0,05 indicated a normal distribution. In this way, EuroSCORE I, EuroSCORE II, and STS score presented asymmetric distributions and were analyzed by univariate non-parametric Mann-Whitney U test. Other quantitative variables with normal distributions were analyzed with *t*-test for independent samples. Descriptions of the quantitative variables were made by mean and standard deviation. Qualitative variables were described by absolute number and the related proportion (%). To analyze this kind of variable we applied the two tailed Pearson’s Chi-square.

The baseline characteristics and outcomes were compared according to the study group (on-pump *vs*. off-pump) with the univariate tests previously mentioned. For this analysis, a *P*-value < 0.05 was considered significant. A univariate analysis stratified by the occurrence of death in 30 days was also carried out in order to evaluate and select potential predictors for the occurrence of the outcome. Only in this case, to select the independent variables for the regression model, we considered significant *P*-values < 0.10 in the analysis stratified by death rates.

Based on the univariate analysis, we selected for the regression model the following variables as independent variables: gender, COPD, AF, and preoperative anemia. In addition to these variables, we also used CPB as an independent variable because it is our main stratification variable. Therefore, the regression model had, as a dependent variable, the occurrence of death in 30 days and, as independent variables, gender, COPD, AF, preoperative anemia, and use of CPB. Bearing in mind that our outcome is a dichotomous categorical variable, we used the corresponding multivariate model for the analysis, a binary logistic regression.

## RESULTS

None of the baseline clinical and demographic characteristics showed a significant difference between the groups (Table 1). This demonstrates a high degree of homogeneity between the two groups, obtained through propensity matching technique, allowing for a solid comparison between the 30-day outcomes of isolated CABG.

**Table 1:** Baseline characteristics stratified by CPB support.

Characteristics	Off-pump CABG (n=332)	On-pump CABG (n=332)	*P*-value
Female gender	98 (29.5%)	108 (32.5%)	0.402
Age (years)	62,7±9,8	62,3±8,6	0.568
Hypertension	276 (83.1%)	270 (81.3%)	0.542
Diabetes	128 (38.6%)	138 (41.6%)	0.428
AMI	109 (32.8%)	109 (32.8%)	1.000
Renal impairment	32 (9.6%)	33 (9.9%)	0.986
Hemodialysis	08 (2.4%)	12 (3.6%)	0.364
Creatinine clearance	75,1±27,2	77,3±29,9	0,333
Smoking	67 (20.2%)	67 (20.2%)	1.000
COPD	16 (4.8%)	18 (5.4%)	0.725
Stroke	25 (7.5%)	24 (7.2%)	0.882
PVD	14 (4.2%)	13 (3.9%)	0.844
Atrial fibrillation	08 (2.4%)	07 (2.1%)	0.794
NYHA class III or IV HF	63 (19%)	61 (18.4%)	0.842
LV ejection fraction (%)	61±12,0	60±12,7	0.199
PASP (mmHg)	29,2±6,6	29,3±7,6	0.957
Frailty	27 (8.1%)	26 (7.8%)	0.886
Anemia	99 (29.8%)	97 (29.2%)	0.865
Instable angina	24 (7.2%)	31 (9.3%)	0.324
Previous CV surgery	6 (1.8%)	3 (0.9%)	0.505
Previous PCI	82 (24.7%)	62 (18.7%)	0.060
Urgency or emergency	9 (2.7%)	7 (2.1%)	0.613
EuroSCORE I	3.34±4.24	3.26±3.34	0.805
EuroSCORE II	1.56±1.71	1.54±1.07	0.808
STS score	0.98±1.01	1.08±1.07	0.202
Complete revascularization	321 (96.7%)	320 (96.4%)	0.832

AMI=acute myocardial infarction; CABG=coronary artery bypass grafting; COPD=chronic obstructive pulmonary disease; CPB=cardiopulmonary bypass; CV=cardiovascular; EuroSCORE=European System for Cardiac Operative Risk Evaluation; HF=heart failure; LV=left ventricular; NYHA=New York Heart Association; PASP=pulmonary artery systolic pressure; PCI=percutaneous intervention; PVD=peripheral vascular disease; STS=Society of Thoracic Surgeons

**Fig. 2:**
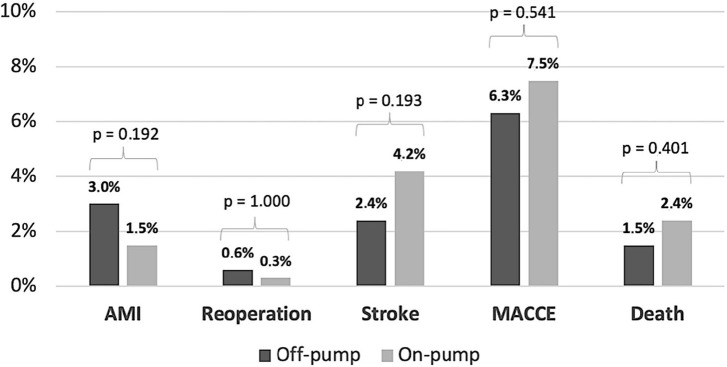
Thirty-day outcomes rates stratified by cardiopulmonary bypass support. AMI=acute myocardial infarction; MACCE=major adverse cardiovascular and cerebrovascular events.

None of the analyzed outcomes showed any differences between the groups, including AMI (3.0% *vs*. 1.5%; *P*=0.192), stroke (2.4% *vs*. 4.2%; *P*=0.193), major reoperation (0.6% *vs*. 0.3%; *P*=1,000), major adverse cardiovascular and cerebrovascular events (MACCE) (6.3% *vs*. 7.5%; *P*=0.541), and death (1.5% *vs*. 2.4%; *P*=0.401). All the major outcome comparisons are shown in Figure 2. The overall mortality rate was 2.0%.

It was possible to establish, through regression analyses, that the use of CPB was not an independent predictor of risk for the occurrence of death (odds ratio [OR]: 2.052; 95% confidence interval [CI]: 0,609 - 6.913; *P*=0.246). Furthermore, other variables with univariate association with 30-day mortality were independent predictors for the outcomes — gender (OR: 4.659, 95% CI: 1.375 - 15.787; *P*=0.013), COPD (OR: 5.903, 95% CI: 1.316 - 26.469; *P*=0.020), preoperative AF (OR: 9.550, 95% CI: 1.507 - 60.509; *P*=0.017), and preoperative anemia (OR: 4.150, 95% CI: 1.272 - 13.541; *P*=0.018) (Table 2).

**Table 2:** Logistic regression analysis.

Variables	B	SE	Wald	P	OR	95% CI
Gender (male)	1.539	0.623	6.109	0.013	4.659	1.375 - 15.787
COPD (yes)	1.775	0.766	5.378	0.020	5.903	1.316 - 26.469
Atrial fibrillation (yes)	2.257	0.942	5.738	0.017	9.550	1.507 - 60.509
Anemia (yes)	1.423	0.603	5.563	0.018	4.150	1.272 - 13.541
CPB (yes)	0.719	0.620	1.345	0.246	2.052	0.609 - 6.913
Constant	-6.050	0.826	53.586	< 0.001	0.002	....

B=unstandardized regression weight; CI=confidence interval; COPD=chronic obstructive pulmonary disease; CPB=cardiopulmonary bypass; OR=odds ratio; SE=standard error

## DISCUSSION

This study adds to our knowledge on the outcomes of on- *vs.* off-pump CABG procedures in Southern Brazil. It shows that there are no significant differences between on- and off-pump CABG mortality and morbidity. In addition, a logistic regression adjusted model did not predict that CPB support is a risk factor for 30-day post-surgery mortality rates in this sample.

These findings are congruent with findings from Lamy et al.^[9]^ (2012) that showed no differences between on- and off-pump isolated CABG mortality and morbidity outcomes within the CORONARY trial study, an international multicenter randomized controlled trial. Mortality rates in the CORONARY trial study were of 2.5% in both on- and off-pump groups, which was similar to our study, especially in the on-pump group, however our off-pump group showed slightly lower rates of mortality (1.5%).

Another large multicenter randomized controlled trial study, called ROOBY trial^[8]^, also showed a lack of significant differences between on- and off-pump CABG procedures. Like in the CORONARY trial, this trend was similar to our results, however, the 30-day mortality rates observed in the ROOBY trials were higher than those observed in our study (7% off-pump and 5.6% on-pump).

The observed differences in mortality rates may be due to the large number of centers involved in data collection and possible heterogeneity in training among the surgeons who performed the surgeries in those large trials^[7-9]^. For instance, the present study involved only one center, with four very experienced surgeons (an average of 15 years of experience for both techniques), who had previously performed at least 250 surgeries of each procedure (on- and off-pump).

Large randomized controlled trials are very difficult to be done, especially in developing nations, with limited research resources, like Brazil. The propensity matching score model allows for retrospective analyses of surgical outcome data that can be compared to RCT results done in large multicenter studies. Previous studies using propensity matching scores in CABG outcome data have been successful in retroactively evaluating mortality and morbidity in on- and off-pump CABG outcomes^[15,16]^.

While Bakaeen’s^[19]^ study had a similar rate of off-pump procedure (18-24%) as compared to our study rates of off-pump surgery (22.5%), Brewer et al.^[15]^ showed lower rates of off-pump procedures (9%). These studies analyzed 1:1 matching of both procedures and indicated similar results. These results highlight that our center in Southern Brazil has produced similar outcomes as international studies in terms of mortality and morbidity, while using a propensity matching score. Our results are relevant as they shed light on Brazilian CABG outcomes in a large scale.

On the other hand, in a cohort of the State of New York (United States of America) with 49,830 patients, a proportion of off-pump surgeries of 27.8% was verified. It was slightly higher than that verified in our study. Using a methodology that used propensity score matching, the researchers found significantly lower rates of mortality and complications associated with short-term off-pump surgery^[20]^.

In the past, we had few Brazilian studies comparing on- and off-pump CABG surgeries. In 2004, Lima et al.^[12]^ published the results of an analysis of 73 Brazilian octogenarian patients. The researchers observed for on-pump CABG patients a surgical mortality rate equal to 11.5%, while the off-pump patients had a surgical mortality rate equal 2.1%. In another study by the same group of researchers, Sá et al.^[13]^ (2010) showed the results of on- and off-pump CABG in a cohort with 941 women. The surgical mortality rate for off-pump CABG was lower when compared with the on-pump technique (3.1% *vs*. 5.3%), but without statistical significance. These two studies were performed considering specific patient characteristics (age or gender), while our study encompassed all patients of the center. Thus, both studies have important information about Brazilian patients, and demonstrated different trends in relation to mortality compared to our study - *e.g*., lower mortality rate for off-pump CABG, compared to on-pump. In 2012, Cantero et al.^[14]^ published the results of a comparison between the two techniques using a cohort with 177 patients. The researchers verified that the mortality rate was similar between the techniques. However, postoperative AMI rates were higher in the on-pump CABG group (7.6%, off-pump; 12.9%, on-pump). In our study, we did not observe this same pattern, and, in addition, AMI rates were much lower (3.0%, off-pump; 1.5%, on-pump).

Finally, we were able to match 664 patients and obtained two very similar groups. In this way, it was possible to compare the 30-day outcomes for the two surgical techniques of revascularization (off-pump and on-pump) more effectively. The propensity score matching is a way of emulating a randomization process, and it raises the level of evidence generated through a cohort study. Our initial cohort had 1,767 patients, and, with the matching, 1,103 patients were discarded from the analysis. This reduction in the number of individuals in the sample is part of the strategy of the propensity score matching, in which a larger number of participants is neglected so that very similar pairs of patients are formed. We think that the confounding results from different studies on the outcomes of the two techniques remains, thus more studies with similar methodologies or RCTs are needed in different populations, as our results contribute to shed light on the characteristics of our population and on the surgical results obtained with CABG procedures in Brazil.

### Limitations

Although all patients were operated on by the same group of surgeons and underwent the same pre and postoperative care protocols, the study was carried out at a single institution. Thus, it is likely that our study represents, in some way, only the population of Southern Brazil. Due to the vast Brazilian territory, there are several different regions, and, as a result, we have an important heterogeneity in healthcare structures and also in the prevalence of cardiovascular risk factors. Another important point, addressed at the end of the discussion, is the reduction in the number of individuals in the analysis with the application of propensity score matching, which may neglect patients with unique characteristics. However, this is a necessary action for obtaining fully balanced study groups. In this way, the comparison of outcome rates can be performed more safely. However, even with the use of this statistical technique that makes the evidence generated from a cohort more reliable and robust, the study does not have the level of evidence from an RCT.

## CONCLUSION

After analysis by propensity score matching, it was possible to observe that patients who underwent surgery with and without CPB had similar incidences of mortality, AMI, stroke, major reoperation, and MACCE in the 30 days post-CABG. It was also possible to verify that the use of CPB was not an independent risk predictor for the occurrence of 30-day mortality. In addition, we could observe that gender, COPD, preoperative AF, and preoperative anemia were independent risk predictors for the occurrence of post-CABG 30-day mortality.

**Table:** 

Authors' roles & responsibilities	
AR	Substantial contributions to the conception or design of the work; or the acquisition, analysis, or interpretation of data for the work; drafting the work or revising it critically for important intellectual content; agreement to be accountable for all aspects of the work in ensuring that questions related to the accuracy or integrity of any part of the work are appropriately investigated and resolved; final approval of the version to be published
GC	Substantial contributions to the conception or design of the work; or the acquisition, analysis, or interpretation of data for the work; final approval of the version to be published
PN	Substantial contributions to the conception or design of the work; or the acquisition, analysis, or interpretation of data for the work; final approval of the version to be published
BSH	Substantial contributions to the conception or design of the work; or the acquisition, analysis, or interpretation of data for the work; final approval of the version to be published
EL	Substantial contributions to the conception or design of the work; or the acquisition, analysis, or interpretation of data for the work; final approval of the version to be published
MS	Substantial contributions to the conception or design of the work; or the acquisition, analysis, or interpretation of data for the work; final approval of the version to be published
FLL	Drafting the work or revising it critically for important intellectual content; final approval of the version to be published
FL	Agreement to be accountable for all aspects of the work in ensuring that questions related to the accuracy or integrity of any part of the work are appropriately investigated and resolved; final approval of the version to be published
